# Assessing Liver Function in Rat Models of Acute Liver Failure Using Single-Photon Emission Computed Tomography and Cytokine Levels

**DOI:** 10.1371/journal.pone.0323531

**Published:** 2025-05-07

**Authors:** Long Xie, Liqun Huang, Xueting Fang, Jinshun Zha, Yingrui Su

**Affiliations:** 1 Department of Nuclear Medicine, The Second Affiliated Hospital of Fujian Medical University, Quanzhou, Fujian, China; 2 Department of Pathology, The Second Affiliated Hospital of Fujian Medical University, Quanzhou, Fujian, China; Babasaheb Bhimrao Ambedkar University (A Central University), INDIA

## Abstract

**Objective:**

To evaluate liver function using dynamic hepatobiliary single-photon emission computed tomography (SPECT) in different rat models of acute liver failure.

**Methods:**

Twenty-four 6–8-week-old male Sprague–Dawley rats (weight 190–200 g) were evenly divided into four groups. Acute liver failure was induced by intraperitoneal injection of D-galactosamine (D-GalN, 600 mg/kg) and lipopolysaccharide (LPS, 10 µg/kg), common bile duct ligation surgery, and removing 70% of the liver mass. The fourth group served as the control without intervention. The time-activity curves for the liver and heart were generated from dynamic SPECT scans with 99mTc-ethylene hepatobiliary iminodiacetic acid (EHIDA). Image-derived functional parameters (5-minute heart/liver index [HLI5] and 15-minute receptor index [LHL15]) were calculated. Furthermore, correlations of image-derived parameters with serum interleukin-6 (IL-6) levels, liver aspartate aminotransferase (AST) and alanine transaminase (ALT) levels, and liver mRNA expression levels of tumor necrosis factor-α (TNF-α) and chemokine ligand-10 (CXCL-10) were analyzed.

**Results:**

All animals in the experimental groups exhibited varying degrees of liver damage. The SPECT images and indexes (HLI5 and LHL15) of the experimental groups significantly differed from those of the control group (*P* < 0.05). In the experimental groups, serum IL-6 levels and liver mRNA levels of TNF-α and CXCL-10 were significantly higher, while liver AST and ALT levels were significantly lower than those in the control group (*P* < 0.05).

**Conclusion:**

Using SPECT with 99mTc-EHIDA, along with the calculated indexes and levels of various cytokines, presents a dependable method for assessing liver function.

## 1. Introduction

Acute liver failure is a severe consequence of abrupt hepatocyte injury from diverse causes. It has a rapid onset that can evolve rapidly within days or weeks to a lethal outcome. The most prevalent causes of acute liver failure are ischemia, drug-induced liver injury, hepatitis B virus, and autoimmunity [1–3]. The pathology of acute liver failure often shows widespread hepatic necrosis and apoptosis. The clinical manifestations include progressive jaundice, hepatic encephalopathy, and disturbed coagulation. Acute liver failure has a mortality rate as high as 80% [1]. The most common causes of death in patients with acute liver failure are cerebral edema, sepsis, and multisystem organ failure [4–6]. The prognosis of acute liver failure is poor. Therefore, it is of paramount importance to evaluate liver function and commence management promptly. In clinical practice, liver function is usually evaluated using methods such as liver biochemical tests, Child–Pugh classification, and indocyanine green clearance tests. Although these methods have value in a certain aspect of liver function assessment, their clinical development and effect are limited due to the narrow scope of assessment, cumbersome determination methods, the need to purchase special instruments or equipment, and the existence of liver toxicity of drugs. Given the limited assessment scope regarding acute liver failure and tedious measurement protocols, there is a pressing need to develop a more direct, comprehensive, and precise method, such as an imaging method, to assess hepatic function. In recent years, hepatobiliary scintigraphy has been increasingly recognized as an effective method for assessing hepatic function [7–9]. In recent years, various researchers have utilized imaging agents such as ^111^In-octreotide, ^99m^Tc-sestamibi, and ^99m^Tc-mebrofenin to evaluate liver function through SPECT. Although some progress has been made, the scope of evaluation remains limited, mostly focusing on surgical cases, healthy populations, or animals. Further research is needed to explore its broader clinical applications [10–12].

Given that previous studies have often focused on a single liver injury model [7,8], we intended to explore more causes of liver injury, so we established separate experimental models for the D-GalN + LPS, partial hepatectomy, and common bile duct ligation. As research on dynamic hepatobiliary scintigraphy grows [7,8], its application in assessing liver function gains increasing attention. However, its precise value in evaluating liver function and its correlation with changes in liver function remain unclear. Furthermore, systematic animal studies are required to validate the efficacy of dynamic hepatobiliary scintigraphy in measuring liver function with visual interpretation of the images and several quantitative functional parameters, such as 5-minute heart/liver index (HLI5) and 15-minute receptor index (LHL15). These nuclear medicine quantitative evaluation indicators have a good correlation with Child–Turcotte criteria score to evaluate the degree of liver function injury [13,14].

Previous studies have shown that proinflammatory cytokines such as interlukin-6 (IL-6), TNF-α, and CXCL10 are responsible for certain pathological manifestations of hepatocyte damage [15–17]. In the present study, we induced acute liver failure using methods corresponding to the common different clinical causes of liver damage, conducted dynamic hepatobiliary single-photon emission computed tomography (SPECT) imaging, and measured the levels of inflammatory cytokines, aiming to elucidate the liver damage mechanisms and provide clinical guidance for diagnosis and treatment. Ultimately, we aimed to establish a comprehensive method including quantitative imaging tools and related cytokines to assess liver function by studying different rat models of liver injury, and thereby reduce the incidence of liver failure and improve the prognosis in patients with acute liver damage.

## 2. Methods

### 2.1 Reagents

Reagents used in this study included the following: 99mTc-EHIDA (Catalog No. 20231228, JiangYuan Industrial Technology and Trade Corporation, Wuxi, China); D-GalN (Catalog No. JO612A, Meilun Bio, DaLian, China); LPS (Catalog No. MB5L98-1, Meilun Bio, DaLian, China); acetaminophen (Catalog No. B353231337769, Meilun Bio, DaLian, China); alanine aminotransferase assay kit (Catalog No. C009-2–1, Jiancheng Bioengineering Institute, Nanjing, China); aspartate aminotransferase assay kit (Catalog No. C010-2–1, Jiancheng Bioengineering Institute, Nanjing, China); hematoxylin and eosin staining kit (Catalog No. C0105S, Beyotime Biotechnology, ShangHai); rat IL-6 enzyme-linked immunosorbent assay (ELISA) kit (Catalog No. MM-0190R2, Meimian Industrial, Jiangsu, China); RNAiso Plus (Catalog No. 9109, Takara Biomedical Technology, Beijing, China); Plus All-in-One 1st Strand cDNA Synthesis SuperMix (Catalog No. E047-01A, Novoprotein Scientific, SuZhou, China); SYBR Quantitative Polymerase Chain Reaction (qPCR) SuperMix Plus (Catalog No. E096-01B, Novoprotein Scientific, SuZhou, China); isoflurane (Catalog R510-22, Ruiwode Life Science, Shenzhen, China).

### 2.2 Equipment and instruments

The equipment and instruments used in this study included Symbia™ T Series SPECT/CT (Siemens Medical Solutions USA, Inc.); desktop refrigerated high-speed centrifuge, automatic tissue hydroextractor, tissue embedding machine, tissue embedding machine with low-temperature control, and slide flattening table with slide dryer from Kuohai Medical Technology Co., Ltd. (Xiaogan, Hubei, China); rotary microtome (Thermo Fisher Scientific, Inc.); real-time quantitative polymerase chain reaction (PCR) system (Applied Biosystem, USA); PCR thermocycler (Bio-Rad, USA); Ultra-Micro UV-Visible Spectrophotometer (BioTeke Corporation, Beijing, China); K3 ELISA reader (DenLey Technology Co., Ltd., Guangdong, China); and mold incubator (Lichen Bang Xi Instruments & Technology Co., Ltd., Shanghai, China).

### 2.3 Animals

#### 2.3.1 Rats.

Twenty-four male Sprague–Dawley rats (body weight 190–200 g) were purchased from SPF (Beijing) Biotechnology Co., Ltd., with license ID SCXK(Jing)2019–0010.

#### 2.3.2 Ethics approval and consent to participate.

The experimental procedures in this study complied with the National Institutes of Health publication (NIH, 86–23, 1985) Guidelines for Use of Laboratory Animals. The study was approved by the Ethics Committee of the Second Affiliated Hospital of Fujian Medical University (reference number: 2021–172) and complied with the ARRIVE guidelines.

#### 2.3.3 Disease models and animal groups.

The 8-week study used 24 rats that were monitored twice daily (or more frequently if signs of distress were noted). All research staff were trained in animal handling, ethical care, and euthanasia to ensure compliance with the relevant animal welfare standards.

The rats were anesthetized in a glass box with a mixture of 2% isoflurane in oxygen (1.0 L/min). Then, a close-fitting face mask was used for continuous anesthesia during liver surgery or SPECT examination. The rats were divided into four groups. One group was kept without intervention (the control group, N = 6). Acute liver failure was induced in three experimental groups using different methods as follows: intraperitoneal injection of D-galactosamine (D-GalN, 600 mg/kg) and lipopolysaccharide (LPS, 10 µg/kg) (N = 6), common bile duct ligation surgery (N = 6), and partial hepatectomy by resection of 70% of the liver mass (N = 6); and the model was established at 48 hours, 72 hours, and 24 hours [18–20] respectively.

### 2.4 Dynamic hepatobiliary SPECT imaging and image analysis

Upon the successful establishment of the acute liver failure models, the rats underwent dynamic hepatobiliary scintigraphy using a SPECT system equipped with a low-energy general purpose collimator. The animals were fasted for 4–6 hours, with ad libitum access to water. Each animal was anesthetized and secured on the scanner bed in a supine position. The rats were 2 cm below the anterior SPECT detector, with the body aligned with the center of the probe. For SPECT imaging, each animal was injected with 0.1 mCi/kg 99mTc-ETHIDA through the tail vein immediately after starting the dynamic hepatobiliary scan. The SPECT acquisition parameters were as follows: energy peak of 140 keV, matrix of 128 × 128, 60 seconds per frame, and 15 frames collected. SPECT images were acquired for the control and experimental groups.Two experienced nuclear medicine physicians (Long Xie and Liqun Huang) analyzed the SPECT images and mapped the regions of interest (ROIs) of the rat heart and liver at the same time independently. Radioactivity of the heart and liver 5 minutes after injection (H5 and L5, respectively), and that of the heart and liver 15 minutes (H15 and L15, respectively) after the injection of 99mTc-ETHIDA were used to calculate 5-minute heart/liver index (HLI5 = H5/L5) and 15-minute receptor index (LHL15 = L15/[L15 + H15]) in line with previous studies [21,22]. The average radioactivity values of all ROIs were used for statistical analyses.

### 2.5 Tissue collection and analysis.

Histological and blood biochemical analyses were performed at the same time points as for SPECT. Blood samples were taken from the marginal ear vein, centrifuged at 2500 rpm for 10 minutes, and stored at −80°C freezer for later analysis. All live tissues were snap-frozen in liquid nitrogen and stored at −80°C freezer for further analysis.

The levels of ALT and AST in the serum of rats were determined by an automatic biochemical analyzer and a serum aminotransferase detection kit. Serum levels of IL-6 were examined by ELISA. Subsequently, the liver tissue was divided into two parts. One part was fixed in paraformaldehyde, followed by dehydration, paraffin embedding, sectioning, deparaffinization, and staining with hematoxylin and eosin (H&E). Then, the histological changes of hepatocytes were observed with light microscopy and photographed. A single liver tissue section was randomly chosen from each animal and evaluated semiquantitatively by an experienced pathologist (Xueting Fang). The liver damage was assessed using a scoring system based on the severity of congestion, vacuolar degeneration, and the extent of necrosis, with a maximum score of 4 for each criterion [23,24]. Congestion and vacuolar degeneration were scored on a scale from 1 to 4, with 1 indicating rare, 2 indicating mild, 3 indicating moderate, and 4 indicating severe. Similarly, the extent of necrosis was scored from 1 to 4 based on the percentage of necrotic cells, with 1 indicating trace amounts, 2 indicating <30%, 3 indicating 30–60%, and 4 indicating >60%.

mRNA expression levels of TNF-α and CXCL-10 in the other liver tissue part were determined using qPCR, with GAPDH as the internal control. The primers were synthesized by Fuzhou Sunya Biotechnology Co., Ltd.

The primers for the amplification of the indicated genes were as follows: TNF-α forward, 5’-CTCAAGCCCTGGTATGAGCC-3’ and reverse, 5’-CTCCAAAGTAGACCTGCCCG-3’; CXCL-10 forward, 5’-TGCAAGTCTATCCTGTCCGC-3’ and reverse, 5’-TCTTTGGCTCACCGCTTTCA-3’; GAPDH forward, 5’-ACGGCAAGTTCAACGGCACAG-3’ and reverse, 5’-GAAGACGCCAGTAGACTCCACGAC-3’.

All rats were euthanized upon completion of the study using CO2 inhalation; none died naturally. Euthanasia was performed within 24 hours of meeting predefined criteria (e.g., > 20% weight loss, inability to eat/drink, or severe distress). Efforts to reduce suffering included enriched housing, analgesics, proper nutrition, and anesthesia for invasive procedures.

### 2.6 Statistical analysis

Statistical analysis was conducted using SPSS version 26.0 (SPSS Inc., Chicago, IL). Analysis and visualization were performed using GraphPad Prism 9.0 (GraphPad Software, Inc., La Jolla, CA). Data were expressed as mean ± standard deviation. We used one-way analysis of variance (ANOVA) for comparing means across multiple groups, *t* test for comparing means between two groups, and Pearson test for correlation analysis. We used post-hoc multiple-comparison tests after ANOVA. When variance homogeneity was greater than or equal to 0.05, LSD test was used, and when variance homogeneity was less than 0.05, Dunnett T3 test was used. The reliability of the SPECT parameters (HLI5 and LHL15) was determined by calculating the intra-class correlation coefficient (ICC). The 95% confidence interval of each ICC value was calculated. The degree of intra-class correlation was specified according to the value (poor, below 0.50; moderate, between 0.50 and 0.75; good, between 0.75 and 0.90; and excellent, above 0.9). A *P* value lower than 0.05 was considered statistically significant.

## 3. Results

### 3.1 Dynamic hepatobiliary SPECT

#### 3.1.1 Visual inspection of SPECT images.

**Fig. 1:**
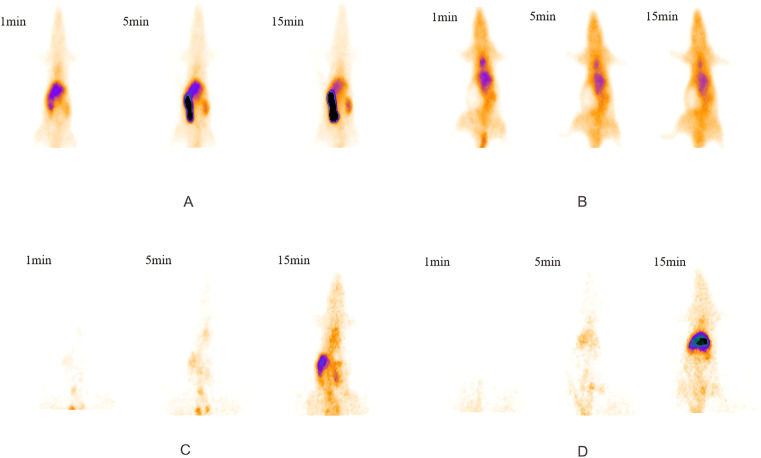
SPECT/CT images of the control group **(A)**, D-GalN + LPS group **(B)**, partial hepatectomy group **(C)**, and common bile duct ligation group **(D)**.

Compared with the control group, all of the experimental groups exhibited a decrease in both the accumulation of 99mTc-EHIDA within the liver and the clearance of 99mTc-EHIDA from the liver. These findings indicated impaired liver function in the experimental groups (Fig.1).

#### 3.1.2 Time–activity curve.

**Fig. 2:**
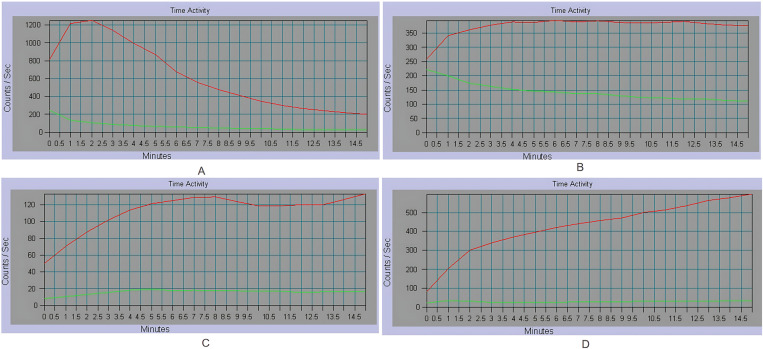
Time–activity curves from dynamic SPECT/CT. **A.** Control group; **B.** D-GalN + LPS group; **C.** Partial hepatectomy group; **D.** Common bile duct ligation group. The red lines represent the count changes of liver ROI, and green lines represent the count changes of heart ROI.

In contrast to the control group, the liver time–activity curves in the experimental groups displayed a lower peak activity of 99mTc-EHIDA, a prolonged time to peak, and a slower clearance. These findings demonstrated gradually impaired liver uptake and excretory functions (Fig.2).

#### 3.1.3 SPECT image–derived indexes.

The values of HLI5 for all animals determined by XL and LQH were 0.16 ± 0.09 and 0.17 ± 0.09, respectively (ICC = 0.911, *P* < 0.05). The values of LHL15 determined by XL and LQH were 0.83 ± 0.08 and 0.83 ± 0.08, respectively (ICC = 0.957, *P* < 0.05). The agreement of measurements of HLI5 and LHL15 was excellent.

**Fig. 3:**
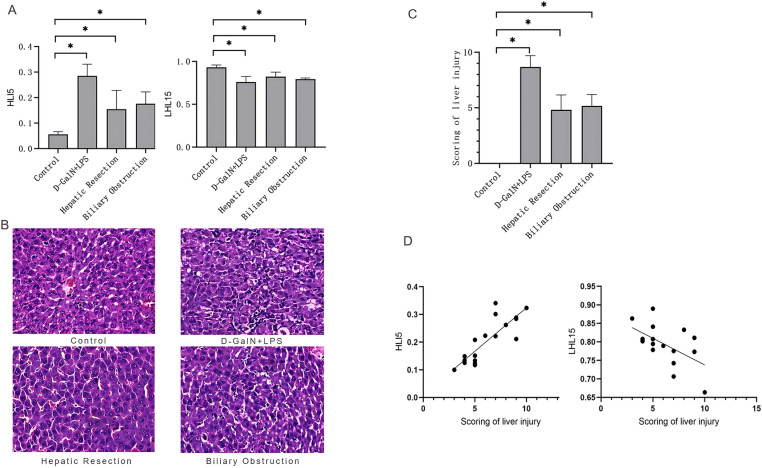
SPECT image–derived indexes and liver histopathology. **A.** The changes in HLI5 and LHL15; **B.** Hematoxylin–eosin staining showing the liver pathology in the control group and the experimental groups (200×); **C.** The liver histopathological scores; **D.** The correlation analysis between the histopathological scores and the SPECT image–derived indexes.

Compared with the control group, with the mean value of 0.06 ± 0.01, all of the experimental groups showed significantly increased HLI5, with the mean values of 0.29 ± 0.05, 0.15 ± 0.07, and 0.18 ± 0.05 for the D-GalN + LPS group (*P* < 0.05), partial hepatectomy group (*P* < 0.05), and common bile duct ligation group (*P* < 0.05), respectively. Compared with the control group, with the mean value of 0.93 ± 0.03, all of the experimental groups showed significantly decreased LHL15, with the mean values of 0.76 ± 0.03, 0.82 ± 0.05, and 0.79 ± 0.01 for the D-GalN + LPS group (*P* < 0.05), partial hepatectomy group (*P* < 0.05), and common bile duct ligation group (*P* < 0.05), respectively (Fig.3A,S1 Table). Additionally, the reference values for the HLI5 and LHL15 parameters in this study were 0.06 ± 0.01 and 0.93 ± 0.03, respectively. We used post-hoc multiple-comparison tests for the HLI5 and LHL15 parameters across the three experimental groups after ANOVA. There was no significant difference observed in HLI5 between the partial hepatectomy group and the common bile duct ligation group (*P* = 0.7889), while significant differences were found among the other experimental groups (*P* < 0.05). Furthermore, there was no significant difference in the LHL15 level among the various experimental groups. The HLI5 in the D-GalN + LPS group was the highest, whereas the LHL15 in the partial hepatectomy group was the highest.

### 3.2 H&E staining results and pathological scoring

#### 3.2.1 Pathological changes.

The liver tissue from each group underwent H&E staining to examine pathological changes. In the control rats, the liver sections displayed normal structure and hepatocyte morphology, without inflammatory cell infiltration, and no apparent abnormalities such as degeneration or necrosis. In contrast, the liver sections from the rats in the D-GalN + LPS group showed disorganized and severely damaged hepatic architecture, along with diffuse hydropic degeneration and extensive hepatocyte necrosis accompanied by diffuse inflammatory cell infiltration. In the partial hepatectomy group, the liver sections revealed hepatocyte necrosis, sinusoidal dilation, hepatocyte hydropic degeneration, some swollen hepatocytes with clear cytoplasm, and a small amount of inflammatory cell infiltration. In the common bile duct ligation group, the liver sections exhibited vacuolar degeneration of some hepatocytes accompanied by varying degrees of inflammatory cell infiltration (Fig.3B).

#### 3.2.2 Pathological scoring of liver damage.

Liver tissue damage was assessed through histopathological scoring of H&E-stained sections. Compared with the control group, which scored 0, the three experimental groups exhibited varying degrees of liver tissue damage, with scores of 8.7 ± 1.0, 4.8 ± 1.3, and 5.2 ± 1.0 for the D-GalN + LPS group (*P* < 0.05), partial hepatectomy group (*P* < 0.05), and common bile duct ligation group (*P* < 0.05), respectively (Fig.3C,S2 Table). There was no significant difference between the partial hepatectomy group and the common bile duct ligation group (*P* = 0.8694), while significant differences were found among the other experimental groups (*P* < 0.05). The pathological scoring in the D-GalN + LPS group was the highest.

### 3.3 Correlation of histopathology and SPECT-derived parameters

The correlation between histopathological scores and the SPECT-derived parameters was analyzed, and the results are presented in Figure 3D. There was a significant positive association between HLI5 and the histopathological score, with a correlation coefficient (r) of 0.8154 (*P* < 0.05), while a negative association was found between LHL15 and the histopathological score, with an r value of −0.5531 (*P* < 0.05).

### 3.4 Measurement of AST and ALT levels

**Fig. 4:**
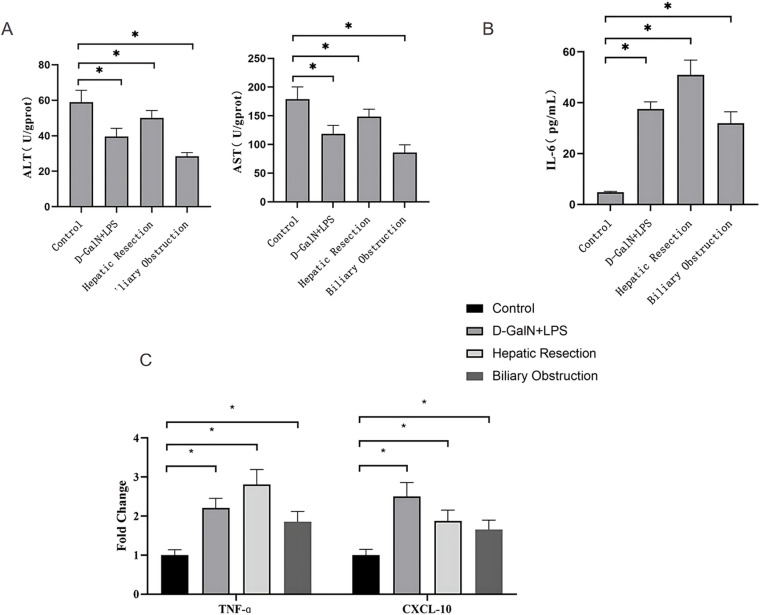
Measurements of liver biochemical parameters. **A.** AST and ALT levels in liver tissue; B. serum IL-6 level; **C.** TNF-α and CXCL-10 expression levels in liver tissue.

We analyzed the levels of ALT and AST in serum of each group of rats. Compared with the control group, the experimental groups exhibited decreased levels of ALT, with the mean values of 58.58 ± 6.78, 39.57 ± 4.59, 50.13 ± 4.17, and 28.47 ± 2.09 (U/gprot) for the control group, the D-GalN + LPS group, the partial hepatectomy group, and the common bile duct ligation group, respectively (*P* < 0.05, for all experimental groups). Compared with the control group, the experimental groups also exhibited decreased levels of AST, with the mean values of 178.77 ± 21.61, 118.59 ± 14.61, 148.69 ± 12.92, and 85.76 ± 13.75 (U/gprot) for the control group, the D-GalN + LPS group, the partial hepatectomy group, and the common bile duct ligation group, respectively (*P* < 0.05, for all experimental groups) (Fig.4A,S3 Table). Except for the partial hepatectomy group and the common bile duct ligation group, there were no differences in the AST level among the experimental groups (*P* = 0.3423). However, ALT and AST levels differed among the experimental groups (*P* < 0.05). The ALT and AST levels in the D-GalN + LPS group were the highest.

### 3.5 ELISA measurements

We measured the serum IL-6 level using ELISA. The serum IL-6 levels in the D-GalN + LPS, partial hepatectomy, and common bile duct ligation groups were significantly higher than those in the control group, with the mean values of 4.81 ± 0.38, 37.56 ± 2.84, 50.94 ± 5.81, and 31.92 ± 4.58 (pg/mL) in the control group, the D-GalN + LPS group, the partial hepatectomy group, and the common bile duct ligation group, respectively (*P* < 0.05 for all experimental groups; Fig.4B,S4 Table). There was no significant difference between the D-GalN + LPS group and the common bile duct ligation group (*P* = 0.0760), while significant differences were observed among the other experimental groups (*P* < 0.05). The IL-6 level in the partial hepatectomy group was the highest.

### 3.6 qPCR measurements

We quantified TNF-α and CXCL-10 mRNA expression levels in liver tissue samples from each group using qPCR. The expression levels of TNF-α mRNA were 1.00 ± 0.14, 2.20 ± 0.25, 2.81 ± 0.38, and 1.85 ± 0.27, and the levels of CXCL-10 mRNA were 1.00 ± 0.15, 2.50 ± 0.36, 1.87 ± 0.28, and 1.66 ± 0.24 in the control group, the D-GalN + LPS group, the partial hepatectomy group, and the common bile duct ligation group, respectively. Compared with the control group, all of the experimental groups showed elevated mRNA levels of TNF-α and CXCL-10 (*P* < 0.05; Fig.4C,S5 Table). Except for the partial hepatectomy group and the common bile duct ligation group, there was no significant difference in the CXCL-10 level among the other groups (*P* = 0.4131). However, TNF-αand CXCL-10 levels differed significantly among the experimental groups (*P* < 0.05). The TNF-αand CXCL-10 levels in the D-GalN + LPS group were the highest.

## 4. Discussion

Acute liver failure is a rare condition with diverse etiologies. It is characterized by extensive hepatocyte necrosis, severe liver dysfunction, and rapid disease deterioration, and often results in high morbidity and mortality [1,25]. Typical clinical workup relies on serum biochemical tests and Child–Pugh classification for early diagnosis and liver function assessment. However, these commonly used methods assess total liver function indirectly and may not always provide pertinent insights, particularly when assessment of regional liver function is crucial [26].

Therefore, there is a need to explore radiological approaches, with nuclear medicine imaging emerging as a promising avenue for directly assessing liver function. SPECT is a commonly used nuclear medicine imaging modality for liver function assessment [27,28]. SPECT has been used widely in clinical practice because of its various imaging agent options, such as 99mTc-GSA, 99mTc-PMT, 99mTc-disofenin, 99mTc-HBS, and 99mTc-EHID [29–31]. 99mTc-diethyl iminodiacetic acid (99mTc-EHIDA) is a widely used SPECT imaging agent in clinical setting. After intravenous injection, 99mTc-EHIDA binds to serum albumin and is then transported to the liver. In the liver, 99mTc-EHIDA dissociates from albumin and is taken up by hepatocytes in the perisinusoidal space. Subsequently, it is secreted into bile through a metabolism process similar to that of bilirubin and finally excreted into the intestine through the biliary system. Using a gamma camera, the dynamic process of 99mTc-EHIDA being taken up by liver cells, secreted into bile ducts, and entering the intestine can be monitored. Due to its rapid blood clearance, high liver uptake, rapid biliary excretion, and minimal interference from bilirubin, it is most widely used clinically for the diagnosis of various liver and biliary system diseases such as persistent jaundice in infants, acute and chronic cholecystitis, postoperative evaluation of the biliary system, post–liver transplantation monitoring, and bile reflux [32–34]. Gamma rays used to form SPECT images may be absorbed and scattered by the patients’ tissue, potentially affecting image quality.

However, SPECT imaging using a more advanced imaging system owned by the unit enables attenuation and scatter correction, which significantly enhances SPECT image quality and spatial resolution. Moreover, a new-style SPECT allows direct and more reliable quantitative assessment of radioactivity uptake in the liver. Even in obese patients or patients with ascites, the quantification of the radioactivity in the liver is not affected by inflammation and congestion [35]. To explore the utility of 99mTc-EHIDA hepatobiliary dynamic SPECT imaging in assessing liver function, we established animal models of acute liver failure corresponding to common clinical causes of liver failure, including drug-induced liver injury, post-hepatectomy, and obstructive biliary injury. Compared with the control group, significantly reduced liver radioactivity uptake was prominently visible in all of the experimental groups on corresponding SPECT images, directly indicating impaired liver function. Our findings are consistent with literature reports [36]. HLI5 in each experimental group showed varying degrees of increase, while LHL15 showed varying degrees of decrease. Histopathological examination of liver sections revealed varying degrees of hepatocyte swelling, necrosis, congestion, and vacuolar degeneration. The histopathological scores of the experimental groups were significantly higher than that of the control group. Furthermore, both SPECT image–derived indexes, HLI5 and LHL15, demonstrated strong correlations with histopathological findings.

ALT and AST are important indicators of liver damage, and the severity of liver damage can be assessed by measuring ALT and AST levels in liver tissue. Our results confirmed that liver damage induced by varying causes led to significantly decreased ALT and AST levels in the liver tissue. During acute liver injury, many cells in the liver die or become very damaged in a short amount of time. Kupffer cells, hepatic sinusoidal endothelial cells, and other cells release various cytokines and biologically active mediators, such as TNF-α, IL-6, and TGF. These mediators can cause further damage to the liver cells through various pathways. Among these cytokines, IL-6 and TNF-α are considered key factors that can increase liver toxicity [37,38]. IL-6 is an inflammatory cytokine that participates in many inflammatory processes in the body. Elevated serum IL-6 often indicates the presence of inflammatory responses [39,40]. Our results showed that the serum IL-6 levels were significantly higher in all experimental groups compared with the control group. IL-6 may play several roles in the pathological process. First, IL-6 activates hepatic endothelial cells and Kupffer cells to release other cytokines, resulting in indirect tissue and cell damage. Second, IL-6 activates neutrophils to produce reactive oxygen species, enhancing the adhesion of neutrophils to endothelium and promoting inflammatory reactions [41], eventually leading to hepatitis, fibrosis, and cirrhosis. Moreover, IL-6 mediates inflammatory immune responses and chronic inflammation, activates JAK tyrosine kinase, and plays a critical role in liver damage [42,43]. In our acute liver failure animal models, TNF-α level in liver tissue increased, suggesting that TNF-α may partake in liver damage. TNF-α is a proinflammatory cytokine secreted by activated monocytes/macrophages. TNF-α enhances the phagocytic function of neutrophils and promotes the differentiation of the inflammatory cells. The rapid increase in blood TNF-α levels causes acute liver injury. TNF-α directly induces cytotoxic effects on liver sinusoidal endothelial cells, resulting in endothelial cell swelling, vessel constriction, and microcirculation dysfunction [44,45]. Additionally, TNF-α impairs mitochondrial function, triggers hepatocyte apoptosis and necrosis, and further exacerbates liver tissue damage [44]. TNF-α and IL-6 synergistically induce the expression of nitric oxide synthase and promote peroxynitrite production, subsequently activating hepatocytes apoptosis pathways and perpetuating a vicious cycle [46]. CXCL10 interacts with its corresponding chemokine receptor, coordinating and mediating the entry of immune cells into damaged or diseased organs [47]. It triggers conformational changes in leukocyte integrins through cascading reactions, facilitating their interaction with adhesion molecules expressed on endothelial cells, thereby promoting leukocyte adhesion and subsequent extravasation. It plays a crucial regulatory role in liver inflammation and hepatocyte necrosis processes [47 ,48]. In our study, the levels of CXCL-10 in the experimental groups were significantly higher than that in the control group, confirming its critical roles in acute liver damage. In our study, the markers including AST, ALT, TNF-α, IL-6, and CXCL-10 in the experimental groups all showed significant differences from those in the control group. This was consistent with the role of SPECT indicators (HLI5, LHL15) in assessing the liver function in this study. Among them, indicators such as HLI5, AST, ALT, TNF-α, IL-6, and CXCL-10 showed significant differences among the experimental groups, while LHL15 showed relatively small differences. These findings have an important reference value for future research.

This study had some limitations. It was conducted in SD rats, and the sample size of each group was small, which may have led to insufficient statistical power and limited the universality of the study results. In addition, the blank control group in this study did not receive any experimental manipulations, and there was no comparison with known effective treatments, so the effectiveness of experimental manipulations or treatments could not be proven. Therefore, in the future experimental design, larger sample size should be adopted, and positive or negative controls should be added, so as to improve the statistical power and reduce the omission of the evaluation of the real effect of experimental interventions. Additionally, considering the differences between different species and humans in drug response and disease pathogenesis, future research should be conducted on animal models that are more similar to humans in biological characteristics to improve the accuracy and relevance of research. C-reactive protein (CRP), which has been seldom used in similar studies in recent years, is an acute-phase protein synthesized by the liver in response to various inflammatory cytokines secreted by various inflammatory cells. As a highly sensitive indicator of systemic inflammation and tissue damage, CRP levels can rapidly reflect changes in a patient’s condition following trauma, inflammation, or infection. Therefore, the CRP measurement can serve as a valuable tool to monitor liver injury, and it should be considered in future experimental designs [49,50]. Finally, we hope that as research advances, the use of SPECT for evaluating liver injury across various models can be applied to clinical practice. This would enable clinicians to assess liver function early, intuitively, and dynamically, thereby improving patient management and outcomes.

## Conclusion

Dynamic hepatobiliary SPECT imaging is a valuable imaging tool for diagnosis and assessing the severity of different types of liver failure. It offers noninvasive and direct monitoring of liver function. SPECT imaging, combined with the calculated indexes and the evaluation of cytokine levels, can become an effective method for assessing liver function of patients with acute liver failure.

## Supporting information

S1 Table Values of HLI5 and LHL15. (XLSX)

S2 Table Pathological scoring. (XLSX)

S3 Table Values of ALT and AST in serum. (XLSX)

S4 Table Serum IL-6 Value by ELISA. (XLSX)

S5 Table qPCR Measurement of TNF-α and CXCL-10 mRNA Levels in Tissue. (XLSX)
